# Optimized complete cytoreduction in ovarian cancer through intraoperative real-time tumor visualization by 5-ALA – a case report

**DOI:** 10.3389/fonc.2023.1288775

**Published:** 2023-12-07

**Authors:** Laura Tascón Padrón, Eva K. Egger, Damian Johannes Ralser, Lucia Otten, Özer-Altan Toksöz, Glen Kristiansen, Walter Stummer, Alexander Mustea

**Affiliations:** ^1^ Department of Gynecology and Gynecological Oncology, University Hospital Bonn, Bonn, Germany; ^2^ Department of Neurosurgery, University Hospital Münster, Münster, Germany; ^3^ Institute of Pathology, University Hospital Bonn, Bonn, Germany

**Keywords:** 5-ala, ovarian cancer, real time imaging, optimazation debulking surgery, intraoperative visualization and guidance

## Abstract

**Introduction:**

Complete macroscopic cytoreduction represents the most important prognostic parameter for overall survival in ovarian cancer. This dogma remains tenacious despite significant improvements in adjuvant systemic treatment. Hence, optimization of surgical therapy is an overarching goal to improve patients’ outcomes. In this context, intraoperative tumor-specific imaging might facilitate optimized cytoreduction. In neurosurgery, intraoperative 5-aminolevulinic acid (5-ALA) guided imaging is applied in clinical routine to assess surgical resection margins. Here, we report the case of a patient with ovarian cancer in whom intraoperative 5-ALA tumor visualization led to optimized complete cytoreduction.

**Objective:**

Intraoperative administration of 5-ALA led to improved complete cytoreduction by identification and resection of additional ovarian cancer tumor manifestations.

**Case:**

The 39-year-old patient, Jehovah`s witness, presented to our department with a left sided ovarian mass, suspicious of ovarian cancer, based on clinical examination, sonographic suspicious features and a CA12-5 elevation. The patient’s medical history and family history was unremarkable. Preoperative CT imaging of the thorax and abdomen showed no pathology besides the adnexal mass. Surgery was performed by a midline laparotomy with hysterectomy, bilateral adnexectomy, pelvic peritonectomy, omentectomy, ureterolysis, diaphragm stripping, adhesiolysis and the collection of peritoneal and rectal samples. Intraoperative 5-ALA imaging using a dedicated excitation and detection loupe system (Reveal, DVI) led to tumor detection at the diaphragm, the omentum and the rectum that was not detectable by palpation and visualization using white light. The pathology results revealed that the 5-ALA positive samples (diaphragm, rectum and omentum) obtained by intraoperative 5-ALA were positive for ovarian cancer.

**Conclusion:**

Intraoperative administration of 5-ALA represents a promising approach to improve complete cytoreduction in ovarian cancer surgery thereby improving clinical outcomes. Hence, further research and clinical trials are required to investigate the potential of intraoperative 5-ALA imaging in ovarian cancer debulking surgery and its impact on long-term clinical outcomes.

## Introduction

Ovarian cancer represents the most lethal gynecologic malignancy. Despite recent significant improvement in systemic treatment, complete cytoreduction remains the most important prognostic factor regarding survival. In high volume centers, complete cytoreduction is achieved in about 63 to 75%. However, 5-year overall survival rates are 45% across all disease stages (1–3). Incomplete cytoreduction might significantly contribute to unfavorable outcomes. In this regard, intraoperative tumor-specific imaging represents a promising approach to optimize surgical therapy by identification and resection of additional ovarian cancer tumor manifestations. Intraoperative imaging with pafolacianin (OTL38), a folic acid analog coupled to indocyanine green, which is the substrate of the folic acid receptor, has proven to be suitable. A recently published phase III trial demonstrated that intraoperative tumor imaging with OTL38, resulted in the detection of ovarian cancer tumor manifestations that would not have been identified or scheduled for resection during standard surgical procedures in >30%. However, the extent to which the additional detection and resection leads to improved overall survival has not yet been determined. OTL38 was approved by the Food and Drug Administration (FDA) in November 2021. There is currently no European Medicines Agency (EMA) approval for Europe.

5-aminolevulinic acid (5-ALA) based intraoperative imaging represents an alternative approach. 5-ALA is a natural amino acid that is metabolized to protoporphyrin-IX through various reactions within the hemoglobin metabolism. In cancer cells, exogenous administration of 5-ALA lead to accumulation of fluorescing protoporphyrin-IX that can be visualized by light at a wavelength of 405 nm and dedicated filters. Intraoperative 5-ALA imaging is well-established in neurosurgery and urology and has proven great potential in ovarian cancer surgery (1–3) and ALA is approved by both the FDA (Gleolan) and EMA (Gliolan) for fluorescence-guided resections of brain tumors.

After administration of 5-ALA, both the eyes and the skin from the patient should not be exposed to strong light sources (e.g. surgical lightning, direct sunlight or intense indoor lightning) for 24 hours. Simultaneous administration of other potentially phototoxic substances (tetracyclines, sulfonamides, etc.) should be avoided. Within 24-hour period following administration of 5-ALA, other potentially hepatotoxic drugs should be avoided.

Commonly observed side effects include vomiting, nausea, elevation of serum bilirubin, transaminases, hypotension, photosensitivity reactions or photodermatoses.

Contraindications for the use of 5-ALA encompass hypersensitivity to the active compound of aminolevulinic acid or porphyrins, acute or chronic manifestations of porphyria and pregnancy.

The publication of data on the application of 5-ALA in gynecological setting is notably scarce (3).

Here, we report the case of a 39-year-old woman who was referred to our center for suspected ovarian cancer. The patient had previously presented to her primary care physician with dysuria. Abdominal ultrasound revealed a large adnexal mass. Due to the patient declaring herself as Jehovah´s Witness and refusing blood transfusion, she was referred to our center for further therapy. Her last gynecological visit was 2018, here a 6cm cyst was observed in one of the ovaries. The patient had no significant medical history, was a non-smoker and had a normal body-mass-index (for patient´s characteristics see **Table 1**).

**Table 1:** Medical history and clinical characteristics of the patient.

History of primary diagnosis and medical history	
**Gender, age**	Female, 38 years
**Staging of primary status**	Bilateral large pelvic mass arising from the ovaries, left ovary 14x10cm, right ovary 7x9cm, perisplenic free fluid, no evidence for distant metastasis
**Tumormarker CA 12-5**	505 U/ml
**Performance Status**	ECOG 0
**Medical history**	None, no history for endometriosis
**Family history**	None
**Gynaecological history**	None, nullipara

In gynecological examination, both ovaries were found to be enlarged, with a big mass that met malignancy criteria. The tumor marker CA 12-5 was elevated and computer tomography staging of the abdomen and thorax showed no suspicion of distant metastatic disease or further intraabdominal spread. Subsequently, cytoreductive surgery with intraoperative pathology evaluation was planned. The patient consented to the procedure as well as the administration of 5-ALA for intraoperative imaging as part of an individual healing attempt.

Four hours prior to surgery, 1,5g 5-ALA was administered orally following the procedure applied in routine neurosurgery (20 mg/kg, 1.5gramms dissolved in 50 ml water; photonamic GmbH & Co., EU/1/07/413/001-003). The patient tolerated 5-ALA well and showed no adverse events.

**Figure 1:**
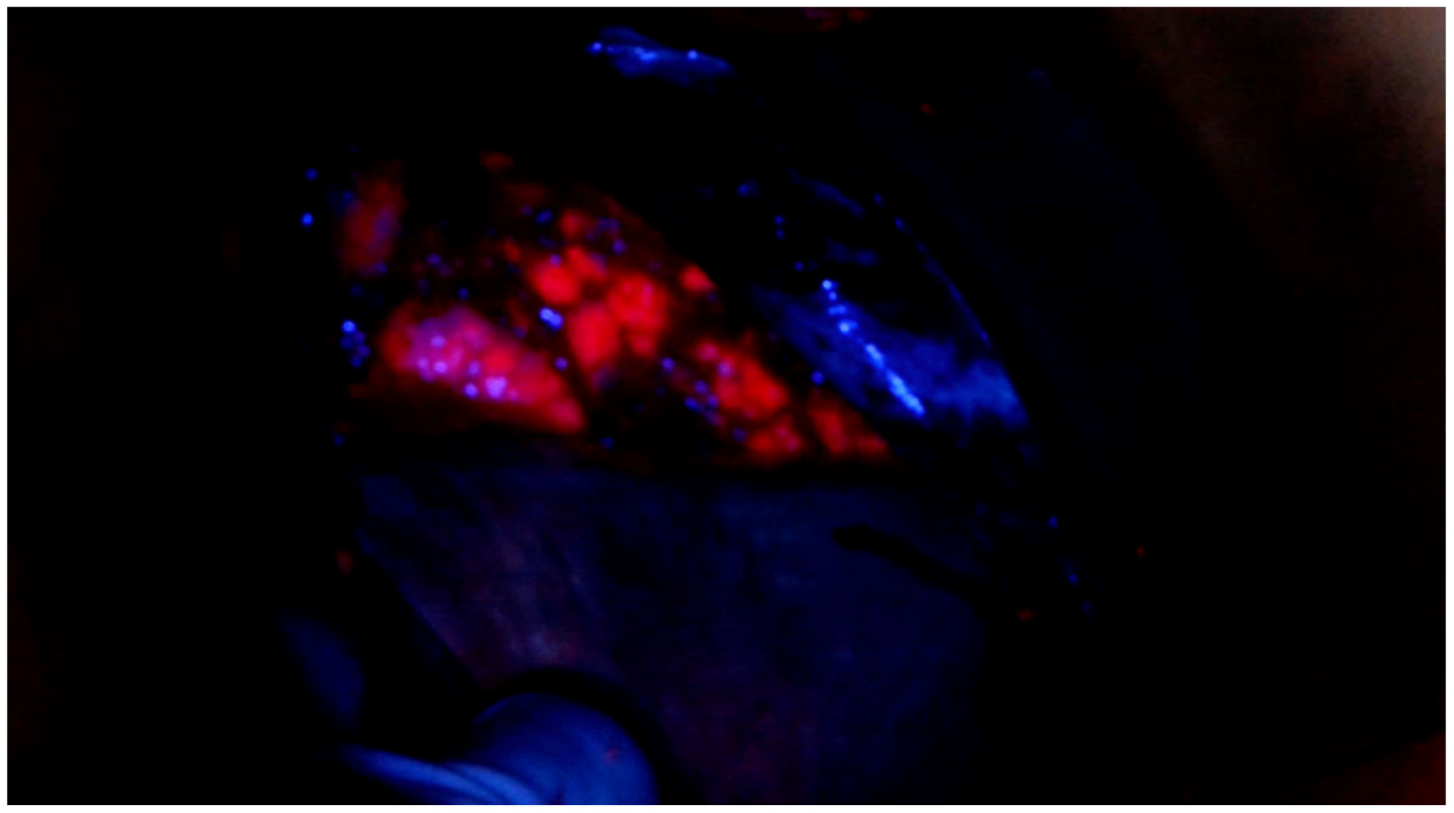
Intraoperative 5-ALA positive detection on the right diaphragmatic peritoneum.

**Figure 2:**
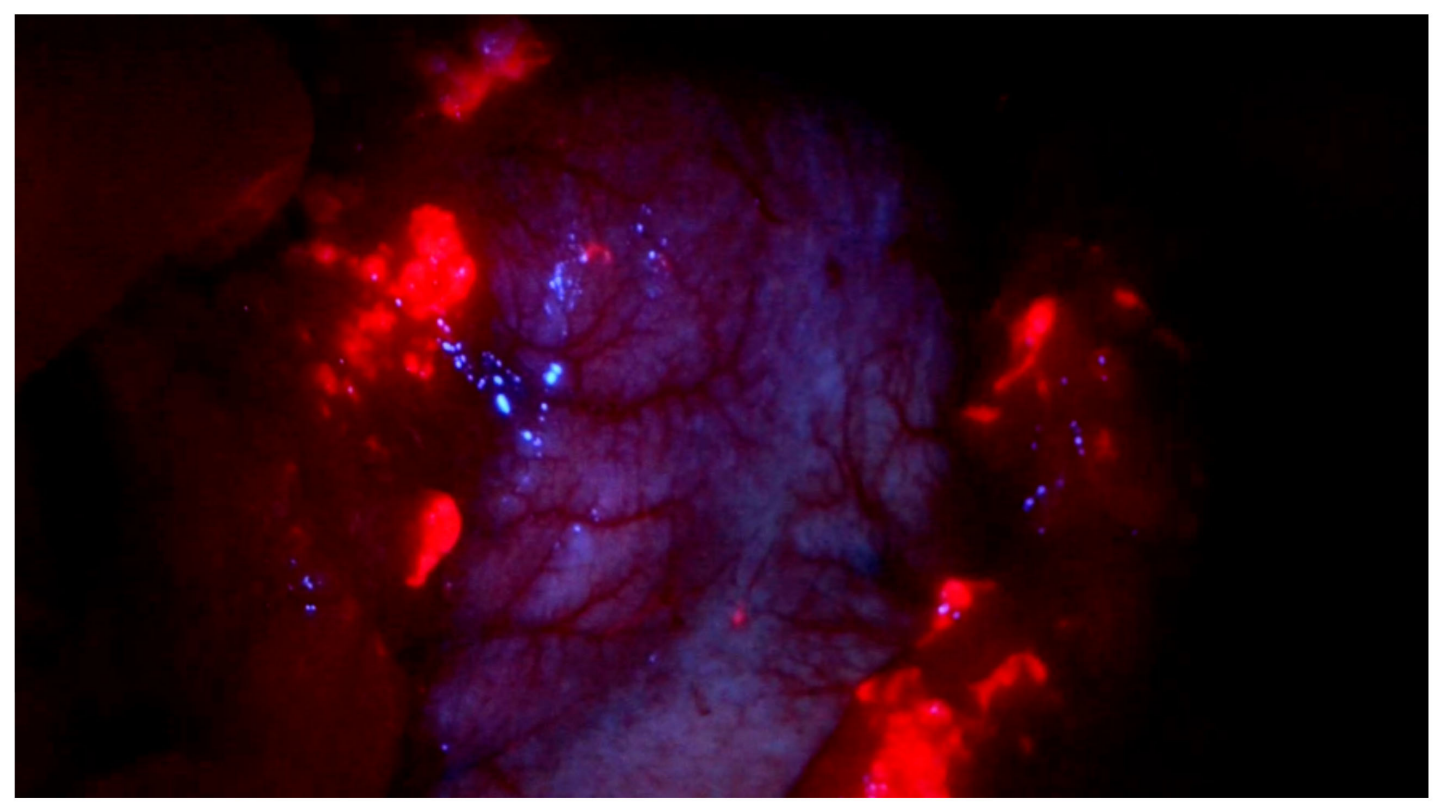
Intraoperative 5-ALA positive detection on the Omentum majus.

A midline laparotomy with hysterectomy and bilateral adnexectomy was performed. Intraoperative pathological counseling (frozen sections) confirmed presence of high-grade ovarian cancer. The procedure was completed with omentectomy and peritoneal sampling. After achieving macroscopic complete tumor resection, 5-ALA imaging was performed by using a fluorescence light source (reveal FGS system). This system enables switching from conventional white light to violet-blue light (405nm), with an additional diode with 450nm providing background detail. 5-ALA positive lesions were detected in the omentum, adjacent to the rectum and on the right diaphragmatic peritoneum. Accordingly, resection of these manifestations was performed (**Figures 1**, **2**). All resected samples were sent for histopathological analysis. The peritoneal cancer index (PCI) was 8. After surgery, the patient recovered appropriately and remained hospitalized for a total of 7 days. No adverse events were recorded.

The histopathological workup revealed diagnosis of a poorly differentiated clear cell adenocarcinoma of the ovary. The samples obtained by 5-ALA imaging were positive for ovarian cancer (see **Table 2**). As a result, the tumor was classified according to TNM as pT3b, pN0, L0, V0, Pn0, R0, G3. According to FIGO-classification it was a Stage IIIb.

**Table 2:** Histopathological results postoperative.

1. **(Left ovary)** Adnexectomy specimen on the left showing infiltrates of a poorly differentiated clear cell adenocarcinoma of the ovary.
2. **(Right ovary)** Adnexectomy specimen on the right, similar to 1, showing infiltrates of a poorly differentiated clear cell adenocarcinoma of the ovary.
3. **(Tumor)** Tumor-free adipose/connective tissue.
4. **(Rectal deposits)** Soft tissue excision with 8 mm infiltrates of the aforementioned clear cell adenocarcinoma.
5. **(Diaphragm)** Soft tissue excision with 2 mm infiltrates of the aforementioned clear cell adenocarcinoma, with older residual bleeding. Tumor-free resection margins.
6. **(Falciform ligament)** Peritoneal carcinosis of a clear cell ovarian carcinoma.
7. **(Rectal deposit)** Peritoneal carcinosis of a clear cell ovarian carcinoma.
8. **(Uterus + Cervix)** Uterus with a maximum 7 mm leiomyoma and regular endometrial mucosa. Serosa with peritoneal carcinosis of a clear cell ovarian carcinoma.
9. **(Bladder peritoneum)** Peritoneal carcinosis of a clear cell ovarian carcinoma.
10. **(Omentum)** Peritoneal carcinosis of a clear cell ovarian carcinoma. Tumor-free resection margins. One tumor-free lymph node (0/1).
11. **(Right colonic gutter)** Peritoneal carcinosis of a clear cell ovarian carcinoma.
12. **(Diaphragmatic and Gerota’s peritoneum)** Peritoneal carcinosis of a clear cell ovarian carcinoma.
13. (**Appendix vermiformis)** Mild acute appendicitis with peritoneal carcinosis of a clear cell ovarian carcinoma.
14. **(Ascites)** Positive tumor cells.

According to local standards, a systemic adjuvant chemotherapy with carboplatin AUC5/paclitaxel 175 mg/m², and bevacizumab 15 mg/kg q3w was initiated. Due to a high genetic risk score of 5, an indication for genetic testing was also performed. The patient showed no evidence of a BRCA mutation.

The patient is presently in a satisfactory condition, exhibiting no evidence of disease progression and has not manifested any complications after the administered chemotherapy.

## Discussion

Complete cytoreduction in ovarian cancer patients prolongs survival more than any other therapeutic tool and will be achieved in experienced centers in about 63% to 75% (4, 5). But 5-year survival rates are as low as 45%, despite advances in systemic therapies in this lethal gynecologic disease. Therefore, microscopic residual disease, in case of complete cytoreduction, not visible or palpable seems likely. Implementing intraoperative molecular imaging shows rates up to 33% of additional tumor tissue not identified by visual inspection or palpation (6).

Here we present the case of an ovarian cancer patient, in whom intraoperative 5-ALA imaging led to optimized complete cytoreduction. 5-ALA imaging resulted in resection of additional ovarian cancer tumor manifestations that were initially not planned for resection based on evaluation by white light and palpation. In this case report the use of 5-ALA led to a larger macroscopic resection of tumor manifestations which avoided a systematic lymphadenectomy and its possible post-surgical complications and long-term side effects.

Intraoperative tumor visualization holds the potential to improve resection rates and subsequently enhance patient prognosis. The ability of real time tumor visualization during surgery facilitates a more sufficient complete removal of malignant tissue. Several studies have demonstrated a clear association between intraoperative imaging and improved surgical outcomes (7–9). This highlights the potential for ovarian cancer surgery.

Currently, there are five FGS agents approved for intraoperative imaging by the American FDA and/or the EMA (10). These include 5-ALA for intraoperative application in patients with suspected high-grade gliomas (9). In ovarian cancer surgery, application of folate conjugated to fluorescein isothiocyanate (folate-FITC) demonstrated increased tumor tissue resection, not visible without this tracer. However, as folat receptor expression represents the biological prerequisite for signal detection, this tracer is not suitable for all ovarian cancer patients (11). A further tracer, OTL38, Pafolacianine, is injected preoperatively and showed to be safe and effective in identifying additional tumor manifestations, invisible prior to near-infrared imaging (10, 11) with identification of up to 33% additional ovarian cancer tumor manifestations (12, 13). Of note, no false negative samples were reported. In study referred to, false positive results were reported in almost 25%, mainly in lymph nodes, omentum and uterine fibroids. One assumption is, that the folate receptor is expressed by macrophages in inflamed tissue as well as by resident macrophages as seen in fibroids, leading to positive fluorescence signals in benign tissue (14).

5-ALA represents an interesting alternative approach, in particular, as no distinct tumoral receptor expression is required. Exogenous administration of ALA-5 leads to a tumor entity-independent protoporphyrie-IX accumulation in tumor cells due to a missing or downregulated ferrochelatase activity in tumors. This feature renders 5-ALA a universally applicable tracer. However, recent data showed heterogeneous response to 5-ALA response in different tumor cells (15). In contrast to OTL38, the strong red fluorescence is readily visible to the eye and does not rely on special infrared cameras and an external monitor for visualizing fluorescence.

It should be mentioned that studies in the past have shown evidence of positive responses in rat models with endometriosis-associated lesions (16). However, it is important to note that this patient had a clear cell ovarian carcinoma, which was not associated with previous endometriosis.

In summary, intraoperative 5-ALA imaging harbors the potential to facilitate optimized complete cytoreduction in ovarian cancer surgery as demonstrated in the reported case.

## Conclusion

Intraoperative real-time tumor imaging holds the potential to optimize the surgical management of ovarian cancer patients which might lead to improved survival rates. However, further systematic studies are needed to explore intraoperative 5-ALA imaging in ovarian cancer surgery.

## Data availability statement

The raw data supporting the conclusions of this article will be made available by the authors, without undue reservation.

## Ethics statement

The studies involving humans were approved by Ethics committee of the University Hospital, Bonn. The studies were conducted in accordance with the local legislation and institutional requirements. The participants provided their written informed consent to participate in this study. Written informed consent was obtained from the individual(s) for the publication of any potentially identifiable images or data included in this article.

## Author contributions

LT: Data curation, Formal analysis, Investigation, Visualization, Writing – original draft. EE: Conceptualization, Methodology, Supervision, Writing – review & editing. DR: Conceptualization, Investigation, Project administration, Resources, Writing – review & editing. LO: Writing – review & editing. Ö-AT: Conceptualization, Software, Writing – review & editing. GK: Visualization, Writing – review & editing. WS: Project administration, Writing – review & editing. AM: Funding acquisition, Project administration, Visualization, Writing – review & editing.
